# A case report and literature review on tocilizumab-cured acute necrotizing encephalopathy caused by influenza A virus

**DOI:** 10.3389/fped.2024.1351478

**Published:** 2024-04-05

**Authors:** YuKun Huang, Bin Zhou, ShaoXian Hong, YaLi Cai

**Affiliations:** Pediatric Intensive Care Unit, Xiamen Children's Hospital, Children's Hospital of Fudan University (Xiamen Branch), Xiamen, China

**Keywords:** acute necrotizing encephalopathy, influenza A virus, cytokine storm, interleukin-6, Tolizumab, children, prognosis

## Abstract

**Introduction:**

Acute Necrotizing Encephalopathy (ANE), is a kind of severe Central Nervous System Disease. The commonest pathogen is the influenza virus. The pathogenesis of ANE is bound up to genetic susceptibility and cytokine storm. Interleukin-6 (IL-6) is deemed as the core function in cytokine storm of ANE and that plays a significant role in evaluating the severity of Influenza-Related ANE. Tocilizumab, an IL-6 antagonist, is known to be safe and effective in the treatment of ANE when used early and has an essential role in improving prognosis and preventing disability.

**Case report:**

This case reports a 2 year 10 month old boy who developed ANE after being infected with influenza A virus (H1N1-2019). After treatment with Tocilizumab, the child's consciousness was clear, no convulsions occurred, the movement of limbs was improved, and the lesions of encephalopathy were significantly reduced.

**Conclusion:**

The early use of Tocilizumab is safe and effective for the treatment of ANE caused by influenza virus.

## 1 Background

Acute Necrotizing Encephalopathy (ANE) is an acute, destructive central nervous system lesion that occurs in various states of infection (1). The clinic is mainly dominated by neurological symptoms such as impaired consciousness, convulsions, and decerebrate rigidity after the rapid onset of fever (2), and complications such as central respiratory failure, shock, and multiple organ dysfunction can result in severe cases (3). About 3–5 million severe cases of ANE are reported worldwide each year, with 290,000–650,000 fatal cases (4); children are susceptible to ANE, with a mortality rate of up to 30% (3), permanent sequelae in 56% (5), and less than 10% complete recovery (6). ANE often develops after viral infections, with influenza virus infections being the most common (1). About 21%–45% of children infected with the Influenza Virus develop neurological symptoms, and the prevalence of ANE is about 0.21% (7).

The injury and prognosis of ANE are intimately related to its pathogenesis. It has been suggested that genetic susceptibility and cytokine storm may be the main mechanisms of injury (8, 9), with interleukin-6 (IL-6)-mediated cytokine storm considered to be the most important (1). Tocilizumab, an IL-6 antagonist (10), has been found to improve the prognosis of ANE with early use of Tocilizumab (11, 12).

This article discusses the pathogenesis of ANE and the mechanism of action and clinical efficacy of Tocilizumab through a case of a child with ANE caused by influenza A virus, who was treated with Tocilizumab and had a positive prognosis, and combined with the study of the literature, to help the early treatment and prognosis of children with ANE.

## 2 Clinical data

The boy, 2 years and 10 months old, was admitted for “fever with vomiting for 1 day”, presenting at the beginning of the disease with high fever, occasional cough, vomiting, diarrhea, and occasional shaking of the limbs. No specific past or personal history.

**Figure 1:**
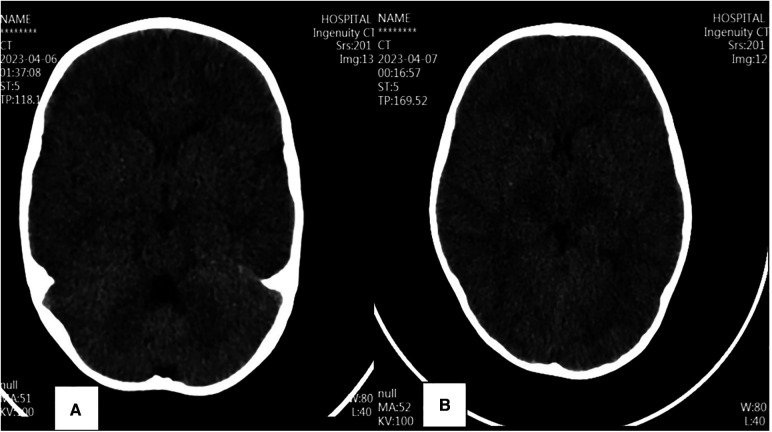
Cerebral CT changes.

Medical examination on admission: Temperature is 39.4 °C, Pulse is 170 bpm, Breathe is 45 bpm, Blood pressure is 86/53 mmHg. Neurological and cardiopulmonary and abdominal examinations showed no significant abnormalities. Pathogenetic testing only suggests a positive antigen for Influenza A Virus (H1N1-2019). Significant abnormalities in Lactate Dehydrogenase, Liver Enzyme, Myocardial Enzyme, Kidney Function (Table 1), Abnormalities in coagulation (Table 2), and remarkably elevated Inflammatory Cytokines (Table 3). Cerebrospinal fluid routine is normal, and Cerebrospinal fluid biochemistry indicates Protein 0.074 g/L, Glucose 4.74 mmol/L, Chlorides 129.6 mmol/L, Lactate Dehydrogenase 11.0 U/L. Immunoglobulin G and complement slightly decreased; absolute T-cell value and absolute NK-cell value slightly decreased; arterial blood gas, blood lactate, and blood ammonia were normal; no obvious abnormality was found in cranial brain CT before hospital admission (Figure 1A).

**Table 1:** Organ function.

	ALB (g/L)	ALT (u/L)	AST (u/L)	LDH (u/L)	CK-MB (u/L)	BUN (mmol/L)	Cr (umol/L)	TG (mmol/L)
04.05	42.1	27.2	62.4	364.8	29.3	8.74	118.1	0.4
04.06	33.9	4,347.2	9,611.4	6,308.4	36.8	7.89	64.2	1.04
04.07	30.6	2,354.3	2,340.5	1,619.3	39.1	6.19	51.8	1.45
04.09	29.3	1,045.3	298.9	355.2	23.7	8.48	52.8	2.79
04.15	38.0	182.6	50.0	432.8	33.6	4.05	14.2	1.8

ALB, serum albumin; ALT, alanine aminotransferase; AST, aspartate amino transferase; LDH, lactate dehydrogenase; CK-MB, creatine kinase-MB; BUN, blood urea nitrogen; Cr, creatinine; TG, triglyceride.

**Table 2:** Coagulation function + PLT.

	PT (s)	TT (s)	APTT (s)	INR (–)	FIB (g/L)	FDP (ug/ml)	D-D (mg/L)	PLT (10 × 9/L)
04.05	15.4	16.4	30.8	1.36	2.60	8.54	1.396	224
04.06	16.2	18.3	34.2	1.43	2.18	24.79	4.108	209
04.07	14.8	19.5	31.9	1.31	2.14	17.57	2.952	174
04.15	10.4	19.5	22.6	0.93	1.69	1.11	0.152	610

PT, prothrombin time; TT, thrombin time; APTT, activated partial thromboplastin time; INR, international normalized ratio; FIB, fibrinogen; FDP, fibrinogen degradation product; D-D, d-dimer assay; PLT, platelets.

**Table 3:** Inflammatory cytokines.

	Fer (ng/ml)	IL-2 (pg/ml)	IL-4 (pg/ml)	IL-6 (pg/ml)	IL-10 (pg/ml)	TNF-α (pg/ml)	IFN-r (pg/ml)	CRP (mg/L)	PCT (ng/ml)
04.06	>1,500	3.32	3.42	3,564.54	119.54	267.06	7.21	28.64	37.71
04.07	445.8	1.98	4.07	27.17	27.19	46.23	2.19	0.94	14.72

Fer, ferritin; IL-2, interleukin-2; IL-4, interleukin-4; IL-6, interleukin-6; IL-10, interleukin-10; TNF-α, Tumor necrosis factor-α; IFN-r, Interferon-r; CRP, C-reactive protein; PCT, procalcitonin.

**Figure 2:**
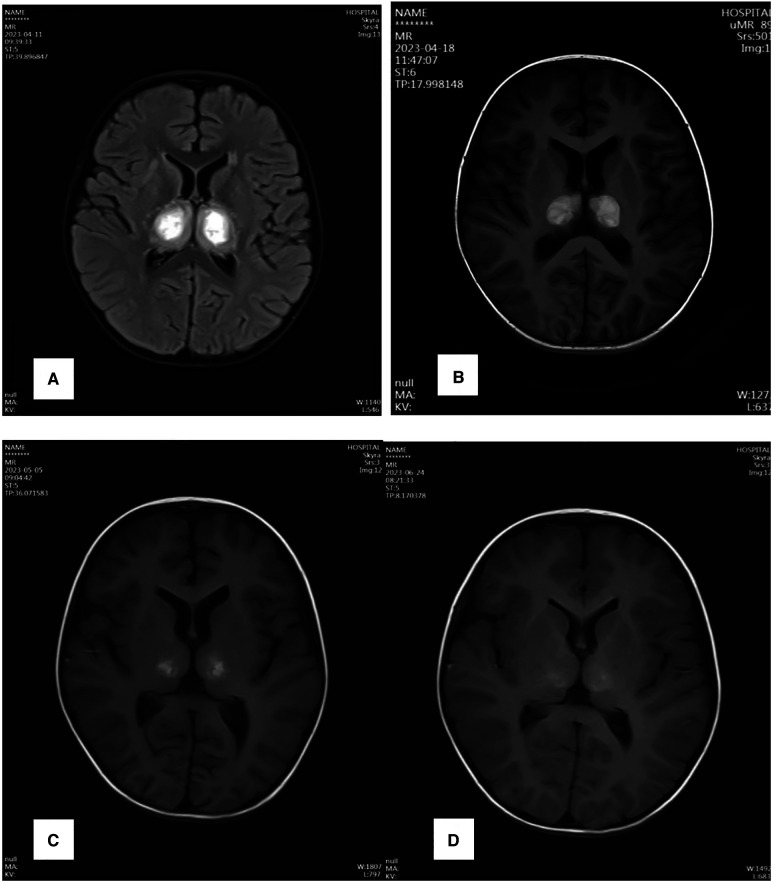
Cerebral MRI changes.

Two hours after admission, the child became comatose, with neck stiffness, showing abnormal in abdominal reflex and cremasteric reflex, meanwhile, hyperactive tendon reflexes, bilateral lower limb ankylosis, and positive pathological signs bilaterally. A review of cranial CT suggested: bilateral basal ganglia and brainstem low-density shadow, considering Necrotizing Encephalopathy (Figure 1A). Electroencephalogram (EEG): Abnormal EEG in a child, with slow background activity and head bursts of slow and sharp waves after wakefulness and sleep phases. The diagnosis of ANE was confirmed, and the child was immediately treated with Tolizumab (12 mg/kg × 1 dose) for anti-inflammation, Mannitol for lowering the cranial pressure, Oseltamivir for anti-virus, organ protection, rehydration, antipyretic and other symptomatic supportive treatments. After 12 h of treatment, the child was in a slight coma, the sense of neck stiffness disappeared, the abdominal reflex could be reacted and weakened, the tibial reflex could not be normal, the tendon reflex was hyperactive, the muscle tone of both lower limbs was slightly weakened, and the pathological signs were positive bilaterally, and the organ function was improved and the inflammation indexes were decreased compared with the previous one (Tables 1–3). After 96 h of treatment, the child had no fever, clear consciousness, cognitive impairment, obvious response to pain stimulation, increased muscle tone in both lower limbs, muscle strength was available, pathological signs were still positive, and cranial magnetic resonance examination suggested that the necrotic lesion had shrunk compared with the previous one (Figure 2A). After 13 days of treatment, the child's limb muscle tone and strength were slightly better than before, the pathological signs were negative, and the cranial magnetic resonance lesion was further reduced compared with before (Figure 2B). Twenty-five days after the onset of the disease, the child could cry, open his eyes on his own, and laugh. One month after the onset of the disease, the child was able to look into the eyes, follow the eyes, turn his head when his name was called, and focus on watching TV. The review of the electroencephalogram showed a normal child's electroencephalogram, with slightly slower background activity, and the cranial magnetic resonance suggested that the lesion had shrunk considerably (Figure 2C). Two months after the onset of the disease, the child was fluent in speech, mobile and cognitively normal. Repeat cranial magnetic resonance showed that the necrotic lesion was further reduced and partially disappeared (Figure 2D).

## 3 Discussion

ANE is an acute, explosive necrotizing lesion of the brain parenchyma (13). It was first proposed by Mizuguchi and other scholars in 1995 (14). ANE has been reported worldwide and can occur in both children and adults (15, 16). The main clinical manifestations of ANE are the rapid onset of neurological symptoms such as impaired consciousness, convulsions, and deafferentation after the onset of fever (2), and in severe cases, complications such as central respiratory failure, shock, multiple organ dysfunction, and diffuse intravascular coagulation can occur (3, 17). The morbidity and mortality rate of ANE in children is more than 30%, and most of them die within the first week after the onset of the disease; about 20% of the survivors develop severe neurological sequelae, and less than 10% of them are completely cured (6, 8). ANE can develop throughout the year, most commonly in the winter months from December to February, and the peak age of onset of the disease in children is from 6 to 18 months of age, and the morbidity rate of men and women is not significantly different from that of race (18). The etiology of ANE is unclear, but it is most often seen following viral infection, and common viruses include influenza virus (type A/type B), herpes simplex virus, human herpesvirus 6, varicella virus, rubella virus, rotavirus, coxsackievirus, measles virus, and microvirus 19 (6, 19, 20). A global outbreak of severe acute respiratory syndrome coronavirus-2 in 2019, cases of SARS-CoV-2 infection leading to ANE occurrence have been reported (21, 22). However, the Influenza Virus is still considered to be the main cause of severe cases and deaths from ANE (1, 5, 23). About 21% to 45% of children infected with influenza viruses develop neurological symptoms, and the prevalence of ANE is about 0.21% (7). Novel influenza A virus (H1N1) is more likely to cause neurological symptoms in children than seasonal influenza (24, 25). In this case, the child's pathogenetic testing was perfected immediately at the onset of illness, and the influenza A virus (H1N1-2019) antigen suggested strong positivity, whereas all other pathogenetic tests yielded negative results, and therefore, the influenza A virus (H1N1-2019) was considered to be the cause of the ANE in this child.

The pathogenesis of ANE is still unclear, and genetic susceptibility and cytokine storm may be the main mechanism of injury (8, 9). Neilson and other scholars found that ANE has an autosomal dominant inheritance characteristic, with an ectopia rate as high as 50% (26), at the same time, they also found that about 70% of patients with ANE have a missense mutation in the RANBP2 gene (ANE type I) (27). RANBP2 is a 358 kDa multi-structural domain cytoplasmic nucleotide that plays a role in facilitating the entry and exit of proteins into and out of the nuclear pore, protein modification, intracellular transport, and energy maintenance (28–30). The available studies have identified missense mutations in the RANBP2 gene at sites such as c.2085C>T, p.Thr653Ile, c.2094A>G, p. Ile656Val, c.2043G>C, p.Trp681Cys, with c.1880C>T, p.Thr585Met being the most reported (31). However, the RANBP2 gene is not the only susceptibility gene for ANE; the Carnitine palmitoyl transferase II (CPTII) gene (32), and the Ephrin type B receptor 2(EphB2) may also be associated with ANE pathogenesis (33). No genetic abnormalities were found in the whole exome genome testing of the child in this case, and hence, genetic susceptibility was not considered in the mechanism of ANE development in this child.

The direct damage of the virus is also considered to be one of the pathogenic mechanisms of ANE. Some studies have found that influenza viruses are neurotropic and can cause neurological lesions by infecting microvascular endothelial cells or entering the central nervous system via the olfactory, vagus, trigeminal, and sympathetic nerves (34, 35). However, no pathogens have been isolated from the cerebrospinal fluid or autopsies of the vast majority of patients (36), and therefore, direct neurological damage from viruses is considered to play a minor role in the pathogenesis of ANE (37, 38). The cerebrospinal fluid of the child, in this case, was negative for pathogenicity, and there were no significant abnormalities in the cerebrospinal fluid routine and biochemistry, which is the same as the conclusion of the study; therefore, direct viral damage was not the causative mechanism of ANE in this child.

Cytokine storm is now thought to be the main mechanism of pathogenic damage in ANE (39). Inflammatory cytokines have been found to be remarkably elevated in patients with ANE complicated by influenza virus infection, and the elevation of IL-6 and Tumour Necrosis Factor-α (TNF-α) was most pronounced (40, 41). *In vitro* and *in vivo* experiments have revealed that IL-6 and TNF-α can cause damage to vascular endothelial cells, disrupt tight junction proteins, and increase the permeability of the blood-brain barrier through the action of trypsin and activation of matrix metalloproteinase 9 (42, 43). After the blood-brain barrier is damaged, cytokines can enter the nervous system and induce apoptosis of neurons and glial cells, leading to brain cell oedema, haemorrhage, and necrosis. It also stimulates the release of more cytokines from glial cells, which affects the function of the nervous system (44). Elevated levels of inflammatory factors increase excitatory glutamatergic neurotransmission in the brain and increase the risk of excitotoxicity, which can cause clinical manifestations such as epilepsy and deafferentation (45). It has been found that during the acute phase of ANE, IL-6 levels in the central nervous system are significantly elevated compared to serum, and that elevated IL-6 levels precede the onset of neurological symptoms (46). High levels of IL-6 are strongly associated with mortality and brainstem dysfunction in patients; therefore, IL-6 is thought to play a central role in the cytokine storm of ANE (1), and serum IL-6 levels can be used to assess the severity of influenza-associated encephalitis disease (47). In this case, the child was perfected inflammatory cytokine testing before the onset of neurological symptoms, and IL-6 and TNF-α were significantly elevated, and then significantly decreased on review during the recovery period, and the magnitude of the rise and fall was more pronounced with IL-6, which is consistent with the manifestation of the cytokine storm of ANE, and it is of great importance in guiding the treatment.

Laboratory tests for ANE include impairment of various organ functions in addition to a significant elevation of inflammatory cytokines such as IL-6 and TNF-α. In this case, the child presented with severe liver and coagulation impairment with significant elevation of serum myosin at the early stage of the disease. It has been found that ANE patients with significantly elevated serum transaminases and albumin in cerebrospinal fluid have a poorer prognosis (48, 49). Scholars found that Creatine kinase-MB >100 U/L, Lactic dehydrogenase >1,000 U/L, hypoalbuminemia, hyperglycemia, hyperuricosuria, and Prothrombin time prolongation and elevated International normalized ratio are all suggestive of an increased risk of death in patients with ANE (3). Therefore, the degree of organ function impairment and coagulation abnormality is an important indicator for assessing the severity of ANE, which is significant in the early identification of ANE critical illness. Blood ammonia was also not found to be abnormal in any of the patients with ANE in most studies (49, 50), which may suggest that there is no significant correlation between ANE and metabolic diseases.

At present, there is no specific treatment for ANE. Controlling temperature in timely, maintaining the airway openly, holding patients calmly, lowering cranial pressure, and taking protective therapy for all organs are the main treatments for ANE. For those infected with the Influenza Virus, early antiviral therapy with Oseltamivir, Zanamivir, or Paramivir has an important role in inhibiting viral replication, but is ineffective in stopping organ damage in ANE (2). This may be related to the continued progression of the cytokine storm in ANE. Some studies have suggested that glucocorticoids and immunoglobulins can improve the prognosis of ANE (3, 15). However, the relevant therapeutic mechanisms have not been elaborated, and glucocorticoids and immunoglobulins lack specificity for the treatment of various critical illnesses, therefore, their significance in the treatment of ANE remains questionable. The significance of plasma exchange therapy in the treatment of ANE has also been continuously explored, but its efficacy is still controversial and needs to be supported by more clinical cases (51).

In recent years, the effective usefulness of Tocilizumab in combating cytokine storms has been continuously reported (11). This is closely related to the role of IL-6 in ANE injury and prognosis (1, 47). IL-6 is a receptor with a soluble (sIL-6R) and membrane-binding site (mIL-6R), which can bind to mIL-6R at low doses or to sIL-6R (trans-signaling) at high doses, thereby binding to the transmembrane proteins (gp130-IL-6-sILr) to produce an activated complex, with signaling mediated by Janus kinase (JAK) and Ras/mitogen-activated protein kinase (MAPK)/NF-κB-IL-6, which promotes B-cell and T-cell differentiation, acute-phase protein production and osteoclast activation (52). High levels of IL-6 have been classified as one of the main features of cytokine storm and Cytokine release syndrome (CRS). IL-6 is also thought to play a crucial role in endothelial cell dysfunction (53). IL-6 stimulates vascular endothelial cells to produce vascular endothelial growth factor, which results in the C5a receptor expression upregulation and increased production of Plasminogen activator inhibitor-1 (PAI-1) on vascular endothelial cells, which promotes increased vascular permeability, blood hypercoagulation (54, 55), in severe cases, systemic inflammation and procoagulant state resulting from organ ischemia, accompanied by associated tissue edema (56). Both of these features are the primary mechanism of action for the massive release of pro-inflammatory cytokines resulting in life-threatening multi-organ damage. Tocilizumab is an IL-6 antagonist that interferes with the sIL-6R and mIL-6R of the receptor, thereby blocking the assembly of the activation complex with gp130-IL-6-sILr, as well as blocking IL-6 trans-signaling (10). Tocilizumab also reduces IL-6 trans-signaling pathway induced PAI-1 production in vascular endothelial cells, thereby reducing organ function impairment (52). Tocilizumab has been found to be important in the treatment of inflammatory responses in patients with Influenza Virus infection (57). Current case reports on ANE treatment suggest that early treatment of ANE with Tocilizumab (weight <30 kg: 12 mg/kg; weight ≥30 kg: 8 mg/kg) is safe and effective, and has an important role in improving prognosis and preventing disability (11, 12). In this case, the child was given Tocilizumab immediately at the onset of the disease, and after 12 h of treatment, the child's clinical symptoms appeared to be significantly improved, the review of organ function damage was significantly improved, the review of inflammatory cytokines decreased significantly, and the most obvious decrease in IL-6, and the follow-up cranial brain imaging suggests that encephalopathic damage was significantly reduced, so it can be assumed that Tocilizumab has a significant role to play in the treatment of ANE. Therefore, it can be considered that Tocilizumab is of great significance in the treatment of ANE. Related studies also found that Tocilizumab has the potential to exacerbate the occurrence of infections during treatment, such as pneumonia, and infections such as herpes zoster, which are the most common (58). However, this was not mentioned in the case of Tocilizumab in the treatment of ANE; moreover, the child, in this case, did not experience any adverse effects during treatment with Tocilizumab.

In summary, ANE, as a serious central nervous system pathology, has a crucial impact on the health and quality of survival of patients. Influenza virus infection is the dominant cause of ANE. Nevertheless, cytokine storm is the main pathogenic mechanism of ANE. IL-6 takes a central role in the cytokine storm of ANE, and the level of IL-6 predicts the severity and prognosis of ANE. Tocilizumab is an IL-6 antagonist, and its early use is safe and effective for the treatment of ANE. In this case, the child with ANE caused by influenza A virus (H1N1-2019) had a positive prognosis and survival after treatment with Tocilizumab, and no notable adverse effects were observed. However, a large number of clinical studies and long-term follow-ups are still needed to confirm the long-term complications after Tocilizumab treatment.

## Data Availability

The original contributions presented in the study are included in the article/Supplementary Material, further inquiries can be directed to the corresponding author.
